# Exploring Cortical Attentional System by Using fMRI during a Continuous Perfomance Test

**DOI:** 10.1155/2010/329213

**Published:** 2009-11-16

**Authors:** M. G. Tana, E. Montin, S. Cerutti, A. M. Bianchi

**Affiliations:** Department of Bioengineering, IIT Unit, Politecnico di Milano, Milan, Italy

## Abstract

Functional magnetic resonance imaging (fMRI) was performed in eight healthy subjects to identify the localization, magnitude, and volume extent of activation in brain regions that are involved in blood oxygen level-dependent (BOLD) response during the performance of Conners' Continuous Performance Test (CPT). An extensive brain network was activated during the task including frontal, temporal, and occipital cortical areas and left cerebellum. The more activated cluster in terms of volume extent and magnitude was located in the right anterior cingulate cortex (ACC). Analyzing the dynamic trend of the activation in the identified areas during the entire duration of the sustained attention test, we found a progressive decreasing of BOLD response probably due to a habituation effect without any deterioration of the performances. The observed brain network is consistent with existing models of visual object processing and attentional control and may serve as a basis for fMRI studies in clinical populations with neuropsychological deficits in Conners' CPT performance.

## 1. Introduction

Sustained attention is defined as the ability to maintain a high vigilance level for a long time, allowing the subject to respond against presentation of infrequent and unpredictable events. One of the most widely used neuropsychological tests for the study of sustained attention is the Conners' Continuous Performance Test (CPT) [1]. The Conners' CPT is derived from the original CPT of Rosvold [2], which was developed to assess vigilance. In the Rosvold CPT, the stimuli are letters which are presented visually one at a time, at a fixed rate and the subject's task is to respond whenever the letter representing the target stimulus appears and to inhibit a response when any other letter appears. The letter chosen as target stimulus is typically X and the Rosenov test is therefore, also known as the “X-CPT.” 

Several neuropsychological tests are derived from the X-CPT and the fundamental paradigm on which all these are based is the serial presentation of target and nontarget stimuli and the subject's task is to respond or inhibit response to infrequent visual target stimuli.

In Conners' CPT, also known as “not-X-CPT”, the target stimuli are the letters X like in the Rosvold's test, but the subject's task is to respond to nontarget stimuli and to inhibit response to target stimuli [3]. Although the Conners CPT is designed to evaluate attention; it has been shown that task performance relies on diverse motor, sensory, and cognitive functions (e.g., maintenance of task instructions, visual perception of stimuli, target identification, and manual response) [4].

For this reason, abnormal performance on the CPT in clinical population may be caused by primary attention deficits or damages to neural systems that are engaged by the task. Therefore, it is of primary importance to identify and localize the brain regions forming the neural networks activated by CPT in healthy subjects to better understand the relationships between performance deficits and developmental or acquired disruption of brain networks in clinical populations [5].

The first aim of our work is to study the neural networks of brain regions involved during the Conners CPT paradigm, by using functional magnetic resonance imaging (fMRI) in order to identify the different contributes to task performance coming from different brain regions.

Another open issue in the investigation of cortical attentional system is understanding the temporal trend of the brain response while performing the CPT task in the fMRI scanner system. The second objective of this work is, therefore, to investigate how fMRI activation in brain regions that are engaged during the performance of Conners' CPT task, evolve during the entire duration of the sustained attention test.

## 2. Materials and Methods

### 2.1. Subjects

Eight right-handed healthy adult volunteers participated in the study, 7 of them are male, and 1 is female with a mean age of 18.8 years (SD 2.9). Participants were recruited among the staff and students working in the “Ospedale Maggiore Policlinico, Milan, Italy” in which the experiment was performed.

All the subjects had normal vision and they had not history of neurological or psychiatric disorders.

### 2.2. Continous Performance Test (CPT)

The task used in the present study is the classical Conner's CPT test. It consists of 26 different letters, of the English alphabet, presented sequentially in random order, on goggle system used by the subject during fMRI scanning procedure. The instructions about the test are given before the test by means of a headphone system. Subjects were asked to press the response button with the forefinger of their right hand as fast as possible when any letter other than X appeared (“Go” event) and to withhold the response when the letter X was displayed (“No-Go” event). Letters were presented with interstimulus interval (ISI) of 1 or 2 s and remained on the screen for 250 milliseconds (stimulus duration). The attention task was 10 minutes long and a total of 450 letters were presented. An initial baseline lasting 2 minutes and a final recovery period lasting 2 minutes were also recorded. During baseline and recovery, meaningless images (differently oriented geometrical lines) were presented to the subjects. 

The CPT test was implemented with the software “Presentation, version 0.81, build” (Neurobehavioral Systems). The package “Presentation” is able to produce the stimulus and to synchronize it with the MR scanner (mripulse function). Moreover, it is possible to obtain a logfile with the onset times of stimuli and the response times of the subject. The logfile information allows to compute the number of omission errors and of commission errors. An omission error is committed whenever the subject does not respond to the nontarget stimulus; a commission error is committed whenever he gives a response to a target stimulus.

### 2.3. Data Acquisition

MRI scans were performed into an 1.5 T scanner (Eclipse Marconi—Philips system) at the Department of Diagnostic and Interventional Neuroradiology, Ospedale Maggiore Policlinico, IRCCS, Milan, Italy, using a standard head coil. Functional MRI images were acquired with an echo-planar imaging (EPI) sequence using axial orientation (TE = 60 milliseconds, TR = 3 seconds, flip angle = 90°). Most of the brain was covered by 26 slices (parallel to the line linking the anterior and posterior commissures CA and CP) obtained with an in-plane resolution of 64 × 64 and 3.97 mm × 3.97 mm × 4 mm voxels. During the Conners' CPT fMRI data were collected in one run of 290 images. The first 10 volumes (30 seconds) were discarded from the following analysis, to allow steady state to be reached. Hence 280 volumes were used: the volumes from 11 to 50 (2 minutes) are the first rest time, the volumes from 51 to 250 (10 minutes) are the active time, the volumes from 251 to 290 (2 minutes) are the second rest time. Duration of the test was approximately 14 minutes.

### 2.4. Data Analysis

**fMRI Data Preprocessing**

The fMRI images were motion corrected and spatially smoothed with a 10 mm × 10 mm × 10 mm full width at half maximum Gaussian kernel by using SPM8b software package (http://www.fil.ion.ucl.ac.uk/). fMRI images were then spatially normalized to the neuroanatomical atlas of Talairach and Tournoux (using a 12 parameter affine approach and a T2* weighted template image).

**First Level Analysis**

At a first stage of analysis fMRI data of each participant were analyzed using the mass univariate approach based on General Linear Model (GLM) Theory implemented in SPM8b.

First level analysis was performed using a block design in which five regressors (length 2 minutes 40 images volume) were used to model the BOLD response during the time in which the CPT task was executed by subjects. The regressors were built by convolving the SPM canonical haemodynamic response function (HRF) [6] with five box car functions of 2 minutes duration each, in such a way to cover the entire duration of the sustained attention test (10 minutes).

The parameters of motion correction resulting by image realignment are also included in the first level design matrix, as unwanted-effect regressors in order to remove residual movement artifacts [7].

The whole set of regressors modeling the effects of interest and the unwanted effects forming the first level design matrix is then fitted to the image data of each subject involved in the experiment. After the estimation of the regression coefficients, inference on relevant contrasts of their estimates was performed using a *t*-Student statistic. A set of first-level *t*-contrasts was specified, each contrast including a weight of one for a particular regressor of interest and a weight of zero for all other regressors. This results in five *t*-contrasts for each participant.

**Second Level Analysis**

At a second stage of analysis, the contrast images obtained at the single subject-level were included into a second level design matrix in order to compute a within-subjects one-way analysis of variance (ANOVA).

The second level design matrix was built by defining a single factor with five levels (each level corresponding to one temporal block of two minutes). Therefore, each level of ANOVA design included the set of first level contrast images from all participants relative to a certain temporal block.

Within the one-way ANOVA design, we computed Student *t*-test obtaining five statistical maps, one for each block of 2 minutes. The spatial *t*-maps so obtained were thresholded for significance both at *P* = .001 without correction for multiple comparison and at *P* = .05 with False Discovery Rate correction.

Regions of interest (ROIs) were defined as clusters of contiguous voxels with a *t*-stat above the threshold. 

In order to investigate how magnitude and extent of activation in brain regions evolve while performing the CPT task, we measured the volume of the ROIs identified for each temporal block of 2 minutes and averaged across subjects the spatial mean of the time courses of the voxels within each ROI.

## 3. Results

Table 1 illustrates the fMRI activations during the first 2 minutes of the CPT task. Brain areas associated with the task performance were located in the cingulate, temporal, and occipital cortical regions and in the cerebellum. BOLD activation in the cingulate cortex is almost entirely located in the anterior division and the greatest brain activation, in terms of volume extent of BOLD response and magnitude (maximum value of T-statistic in the cluster), is located in the right anterior cingulate cortex (ACC).

**Figure 1:**
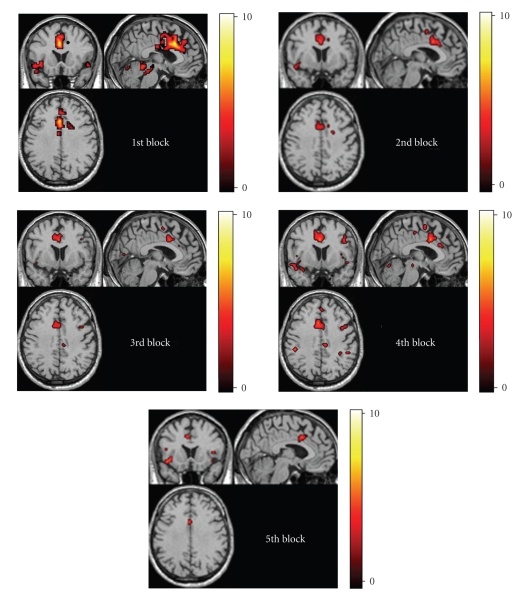
Brain activity during the five blocks of the sustained attention task.

In Figure 1 it is shown how the fMRI activation evolves across the five temporal blocks: comparing with the first block, in the second block (2–4 minutes) and in the third block (4–6 minutes) of the attention test we observed a progressive decreasing of the volume of the activated areas and of the magnitude of their BOLD response. In the fourth block (6–8 minutes), instead, an increasing of the brain activity is observed and in the fifth block (8–10 minutes), there is a new decreasing. 

**Figure 2:**
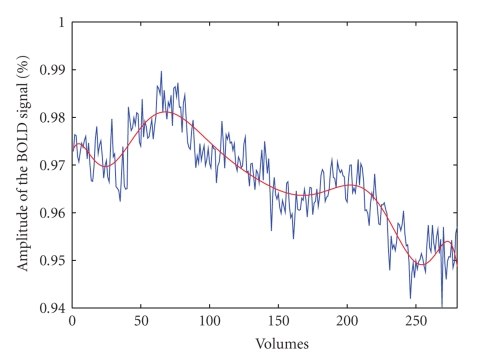
Time course of the BOLD signal of the activated region in the anterior cingulate cortex averaged across the voxels of the ROI and across subjects. The graphics shows the raw signal (blue line) and an interpolated signal by using polinomial fitting (red line). The volumes from 1 to 40 (2 minutes) are the first rest time, the volumes from 41 to 240 (10 minutes) are the active time, the volumes from 241 to 280 (2 minutes) are the second rest time.

Figure 2 represents the time course of the BOLD response in the ACC ROI during the entire duration of fMRI acquisition comprising the baseline period before the beginning of the attention task, the period in which the test was performed, and a final recovery period after the task (2–12 minutes).

In Figure 2 we can observe the initial increasing of the BOLD signal after the beginning of the test (forty-first volume), a subsequent decreasing until about the one-hundred-sixtieth volume, a newly increasing during the 4th block (160–200 volumes) and a decreasing during the 5th block and the final recovery period (201–280 volumes).

For the other areas, not shown in the papers, we observed a similar temporal trend of fMRI response during the test.

**Figure 3:**
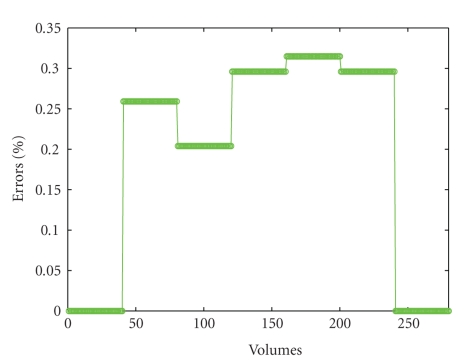
Time course of the number of errors averaged across subjects. The number of errors is expressed in percentage with respect to the number of stimuli.

Figure 3 illustrates the temporal evolution during the test of the numbers of errors committed by the subjects. In each temporal block, we averaged the numbers of errors over each time interval of 2 minutes, and then we averaged the results obtained for each block across the population of healthy subjects. We considered the number of total errors calculated as the sum of omission and commission errors. 

Since in the Conners CPT test, the subject's task is to inhibit the response whenever the letter X representing the target stimulus appears (“No-Go event”) and to respond when any letters appear “Go event,” the omission errors are defined as the lacking responses to “Go-event,” and the commission errors are defined as the responses to “No-Go event.” 

On the basis of the graphic shown in Figure 3 we can observe that the number of errors remains substantially unchanged if we compare the first block (0–2 minutes) and the second one (2–4 minutes), while the number of errors increases in a remarkable way in the third block (4–6 minutes) and in more slightly way in the fourth block (6–8 minutes), and then it decreases in the last temporal block (8–10 minutes). The largest number of errors are committed in the third period of the test, in line with previous clinical literature [8] and with the Conners' C-CPT-II standard [9], which reported a fall in subjects' attentional performance in the middle of the Conners' CPT test.

## 4. Discussion

In this study we identified the network of activated brain regions in a group of healthy subjects by using fMRI during a sustained attention task. This network includes frontal, temporal and occipital and insular areas and the cerebellum. The largest cluster of activation was found in the frontal lobe and, in particular, in the anterior cingulated cortex (ACC). The ACC plays a central role in attentional processing by modulating target selection (i.e., focusing attention) [10], motor response selection [11], error detection, and performance monitoring [12, 13]. 

Activation in left occipital and temporal cortex was located in the ventral visual pathway and it is involved in visual letter recognition as reported in previous studies [14]. The activation of right insular cortex is not documented in previous studies and it is not yet interpreted.

The cerebellum activation confirm results found by the attention study performed by [15], in which it has been shown the lateralization of the attention-related activity to the left cerebellum.

The results relative to the temporal trend of BOLD signal during the sustained stimulation show an initial increasing in fMRI activation in the first part of the test and a subsequent decrease that can be explained by the theory of prolonged stimulation [16], and by the habituation phenomenon that is very frequently encountered in the study of sustained attention [17, 18].

It is widely accepted in literature that brain activity is associated with an increasing in regional cerebral blood flow (rCBF) not matched by a proportional increase in oxygen consumption or cerebral metabolic rate for oxygen (CMRO_2_) [19, 20]. As a consequence of this mismatch, the augmented rCBF requested by brain activity exceeds the augmented oxygen consumption resulting in an initial decreasing of deoxy-Hb and therefore an increasing of the BOLD signal within a few seconds after the onset of stimulation [21, 22].

Therefore, the initial rising of BOLD signal shown in our findings is well explained by this transient pronounced decoupling of rCBF and CMRO_2_. 

After the initial increasing, we observed a slow decreasing in activation until reaching the baseline. This decreasing can be interpreted as a consequence of a restoration of the equilibrium between the cerebral flow and the oxidative metabolism and so the reduction of the BOLD signal could be partially explained by a restored coupling between cerebral flow response and metabolic demand [16].

The further decreasing after the reaching of the baseline is not documented in literature but can be interpreted on the basis of the habituation effect that is widely encountered in the study of sustained attention. 

Since the phenomenon of the habituation, the brain progressively automates the carrying out of the task and gradually reduces the energetic request. This reduction in metabolic demand causes a rapid return of blood flow to basal level not immediately paralleled by the much slower recovery of the metabolic rate for oxygen. This results in a “negative” uncoupling between CBF and CMRO_2_ which could explain our findings. The presence of a “negative” uncoupling has been already proposed in previous studies in order to interpret the reduction of cerebral haemoglobin oxygenation below the basal level immediately after the end of stimulation [16, 23].

The “negative” recoupling is probably prevented by a new increasing of the brain activity that interrupt the habituation phenomenon and is due to a reaction of the subject to a reduction of the behavioral performance.

In particular, the light increasing of the fMRI response observed in the next to last block (6–8 minutes) followed by a decreasing of oxygenation level in the last block of the test (8–10 minutes) is probably related to the immediately previous (4–6 minutes) reduction of the behavioral performance of the subjects resulting in an increasing of the numbers of errors that we observed in the third block (4–6 minutes).

At a behavioral level, it can be supposed that as a consequence of the increasing of the numbers of errors, the subjects make effort to limit the numbers of errors in order to improve their performances. This results in an increasing of the cerebral activity that, in turn, causes an initial rising of the cerebral oxygenation deriving from an initial decoupling of flow and metabolic demand and a following decreasing deriving from the recoupling of haemodynamic response and metabolic request.

After task cessation a further decreasing of the BOLD signal was found in our data followed by a rising of oxygenation level. This is in accordance with the results showed in [16, 23], and can be interpreted as transient deoxygenation resulting from a “negative” uncoupling between the decreasing of the cerebral flow and of the oxygen consumption. 

The incompleteness of the recovery of the BOLD signal after this initial decreasing can be probably due to the shortness of time interval (2 minutes) during which the post-task is recorded. This assertion is suggested by Near Infrared Spectroscopy (NIRS) studies on the sustained attention that documented a complete recovery of the basal cerebral oxygenation level at about 8 minutes after the end of the test, that is, a recovery time longer than that typical of the motor and purely visual stimulation [24].

## 5. Conclusion

This study is aimed to the identification of the cerebral activations patterns during a sustained attention task in a group of healthy subjects. It also attempts to investigate how haemodynamic activation in brain regions involved in fMRI response to a sustained attentive task, evolves during the entire duration of the test and to understand the relationships between the cerebral activation and the behavioral performance.

Using the neuroimaging fMRI method we identified a complex network of brain regions involved in the sustained attention task. The activations are consistent with the existing models of visual object processing and attentional control and the results of clinical literature obtained with other neuroimaging techniques. 

Timing analysis has shown a progressive decreasing of BOLD response during the CPT task due to the habituation effect and an increasing of the activation in the second half of the test probably correlated with the deterioration of the performance that occurs at the middle of the test.

This work can be a starting point for future investigation of the alteration of the pattern and of the dynamical behavior of cerebral network associated to neuropsychological deficits in clinical population. The relationships between specific patterns of activation and behavioral deficits may help us to identify targets for behavioral or pharmacological intervention.

## Figures and Tables

**Table 1:** Summary of BOLD activations during the first block (0–2 minutes, where 0 is the beginning of the execution of the task); *xyz* are Talairach coordinates and Max *t* is the maximum value of the *t*-statistic in the cluster (*P* = .05 FDR corrected).

Region	*x*	*y*	*z*	Max *t*	Volume (mm^3^)
Right Cingulate gyrus (anterior division)	3	17	34	8.16	3204
Left cerebellum	−3	−70	−11	4.89	216
Right insular cortex	39	11	−5	5.09	882
Left insular cortex	−45	8	−5	4.70	297
Left lateral occipital cortex, left middle temporal gyrus	−45	−64	2	1.76	153
Left lingual gyrus, Left intracalcarine occipital cortex	−6	−67	4	3.93	45
Right precentral gyrus, right postcentral gyrus	−6	−16	52	4.03	53
Right supramarginal gyrus, right superior temporal gyrus	63	−34	25	3.87	54
